# Supplementary material for: Content-rich biological network constructed by mining PubMed abstracts
Source: BMC Bioinformatics. 2004 Oct 8;5:147. doi: 10.1186/1471-2105-5-147 (PMC528731; doi:10.1186/1471-2105-5-147)
Supplement: Additional File 2 — The original results of the above study (non-essential files are deleted to keep the file size under the limit set by BMC bioinformatics). [file 1471-2105-5-147-S2.bz2 › chilibotAdditionalFile2/dip40/279ID2498083E1102/html/FOSB_JUN.html]

 


 **FOSB** and **JUN** 
  
Found 189 abstracts in PubMed, retrieved 40.  
 

 What does Google say? 
 PDF only 
| .edu only 

---

**Interactive relationship** (e.g. stimulation, inhibition, etc)

**Stimulatory relationship**- Electrophoretic mobility shift assays using nuclear extracts of Jurkat T cells as well as TCR CD3 restimulated primary human T cells demonstrated specific binding of AP 1  [ **JUN** ] , predominantly composed of c  **Jun**  and  **FosB** , to this sequence.  Ref: 12618758 Oncogene, 2003
- Fra 1, Fra 2,  **FosB** , JunB, JunD, c  **Jun** , and c Fos levels are increased by EGCG treatment, as is AP1  [ **JUN** ]  factor binding to hINV promoter AP1  [ **JUN** ]  site.  Ref: 11698415 J Biol Chem, 2002
- In addition, AP 1  [ **JUN** ]  binding was increased in plasmin treated monocytes, with most complexes composed of JunD, c Fos, and  **FosB** .  Ref: 11389038 Blood, 2001
**Neutral relationship**- Based on in vitro analyses, GnRH appeared to enhance the interaction of JunD,  **FosB** , and c Fos at the GnRHR AP 1  [ **JUN** ]  element.  Ref: 12586760 Endocrinology, 2003
- All cells expressed fos, fra1 and  **jun**  proteins and none expressed  **fosB** .  Ref: 11809534 Cancer Lett, 2002
- **FosB**  is a member of the AP 1  [ **JUN** ]  family of transcription factors which represent important regulators of cell proliferation and differentiation.  Ref: 12602926 Breast Cancer Res Treat, 2003
- **fosB**  was up regulated 20 fold, but only minor effects on  **jun**  variants were observed.  Ref: 12820376 Anticancer Res< MedlineTA>Anticancer Res, 2003
- Under conditions of high rhodopsin availability during exposure to light, Fra 2 and, to a minor degree,  **FosB**  substitute for c Fos and enable light induced AP 1  [ **JUN** ]  activity and thus photoreceptor apoptosis.  Ref: 12766089 Invest Ophthalmol Vis Sci, 2003
- Phosphorylated c  **Jun** , JunB, JunD, and Fra 1, but not c Fos,  **FosB** , or ATF 2, are detected in the AP 1  [ **JUN** ]  DNA binding complex in cells exposed to trivalent arsenicals.  Ref: 12475910 FASEB J, 2003
- These studies demonstrate that a functional AP 1  [ **JUN** ]  site mediates MMP 2 transcription in cardiac cells through the binding of distinctive Fra1 JunB and  **FosB**  JunB heterodimers.  Ref: 12371906 Biochem J, 2003
- Gene expression and nuclear translocation of all the AP 1  [ **JUN** ]  members were induced, under both conditions, with differences in their kinetics, except  **fosB**  mRNA in the Clinostat.  Ref: 12020768 Cell Signal, 2002
- Forty six globes were processed for in situ hybridization with oligonucleotide probes for c fos,  **fosB** , c  **jun** , junB and junD mRNAs, and 60 globes were immunohistochemically analysed using anti c Fos and anti c  **Jun**  antibodies.  Ref: 11825018 Exp Eye Res, 2001
- Here we show that transcriptional activation of an AP1  [ **JUN** ]  regulated gene through the mitogen activated protein kinase extracellular signal regulated kinase pathway is characterized, in intact cells, by a switch from a fra2 junD dimer to a junD  **fosB**  dimer loading on its promoter and by simultaneous recruitment of ERKs, CREB binding protein, and RNA polymerase II.  Ref: 12052862 Mol Cell Biol, 2002
- The Wnt mediated repression of c Fos,  **FosB** , and JunB expression was consistent with a decrease in their binding to an AP 1  [ **JUN** ]  promoter element and decreased target gene transcription.  Ref: 11751871 J Biol Chem, 2002

**Non-interactive relationship** (e.g. studied together, co-existance, homology, etc.)

- Ectopic expression of transdominant negative  **Jun**  mutants strongly reduced CD95L promoter activity and activation induced cell death, confirming the functional significance of  **FosB**  c  **Jun**  binding.  Ref: 12618758 Oncogene, 2003
- There was also a strong potentiation of  **FosB**  and a strong reduction in c  **Jun**  expression.  Ref: 11251193 Brain Res, 2001
- Rat HSCs activated in vitro express JunD, Fra2, and  **FosB**  as the predominant AP 1  [ **JUN** ]  DNA binding proteins, and all three associate with an AP 1  [ **JUN** ]  sequence that is essential for activity of the tissue inhibitor of metalloproteinases 1 TIMP 1 promoter.  Ref: 11337499 J Biol Chem, 2001
- Using DNA protein gel shift assays we determined that nerve growth factor stimulates AP 1  [ **JUN** ]  binding in both PC12 and B5P cells, and identified c Fos,  **FosB** , Fra 1, Fra 2, c  **Jun** , JunB and JunD in AP 1  [ **JUN** ]  complexes.  Ref: 11905996 J Neurochem, 2002
- Immunoblot analyses confirmed that c  **Jun** , JunD, JunB, c Fos, Fra 1, Fra 2 and  **FosB**  immunoreactive proteins were present in whole cell extracts of all antral follicles and midluteal phase corpora lutea as well as granulosa cells isolated from different sized antral follicles.  Ref: 12606371 Biol Reprod< MedlineTA>Biol Reprod, 2002
- In primordial and primary unilaminar follicles, all AP 1  [ **JUN** ]  factors studied except for  **FosB**  were detected in granulosa nuclei.  Ref: 12606371 Biol Reprod< MedlineTA>Biol Reprod, 2002
- In contrast, the AP1  [ **JUN** ]  site mediates an osteoblast specific activation caused by the preferential binding of  **FosB**  to CE1 in osteoblastic cells.  Ref: 12186862 J Biol Chem, 2002
- Within the nucleus, Nrf1 and Nrf2 are recruited to the ARE as heterodimers with either small Maf proteins,  **FosB** , c  **Jun** , JunD, activating transcription factor 2 ATF2 or ATF4.  Ref: 11689285 Cancer Lett, 2001
- Because these two proteins did not give any detectable FRET uponantigen addition, we tethered each protein with a leucine zipper motif c  **Jun**  or  **FosB**  at the C terminus to help the neighborhood of the GFP variants.  Ref: 12463362 Anal Chem, 2002
- Of note, in OA chondrocytes, IL 17 and IL 1beta induced collagenase 3 production through AP 1  [ **JUN** ]  occurred with differential protein complexes IL 17 stimulation resulted in  **FosB**  activation, while IL 1beta stimulated c Fos.  Ref: 12064845 J Rheumatol, 2002
- We have investigated the expression of the AP 1  [ **JUN** ]  transcription factor proteins, fos,  **fosB** , fra1, fra2,  **jun** , junB, junD, using Western blot analysis, in several types of asynchronously proliferating cells.  Ref: 11809534 Cancer Lett, 2002
- Expression of egr 1, egr 2, egr 3, c  **jun** , junB, c fos,  **fosB** , fra 1, fra 2, and CREB genes was analyzed by reverse transcription polymerase chain reaction in Schwann cells isolated from neonatal rat sciatic nerves and in the cell lines MSC 80, NIH 3T3, and CHO.  Ref: 11460264 Glia, 2001
- Among the early response genes analyzed, c myc, junB, junD, c  **jun** , c fos,  **fosB** , fra, as well as max, mad1 4, sin3, only c  **jun**  and fra 2 mRNAs were up regulated after 5D exposure.  Ref: 12648525 J Steroid Biochem Mol Biol, 2003
- We further studied the expression of  **fosB** , c  **jun**  and junB, in the AP 1  [ **JUN** ]  complex.  Ref: 12820376 Anticancer Res< MedlineTA>Anticancer Res, 2003
- This effect was specific because palytoxin had little effect on c  **Jun** , JunB, JunD,  **FosB** , Fra 1, or Fra 2 binding or on overall levels of transcription factor binding.  Ref: 12460732 Toxicol Appl Pharmacol, 2002
- but there was a marked induction of  **FosB**  and Krox 20, a strong repression of c  **Jun** , and no effect or a reduction of the basal levels of JunD.  Ref: 11744254 Neuroscience, 2001
- Thus, our results demonstrate an important novel function for  **FosB**  dimerized with c  **Jun**  in TCR CD3 mediated AICD in human T cells.Oncogene 2 333 1339.  Ref: 12618758 Oncogene, 2003
- Normal lens epithelial cells expressed mRNA signals for junD, but not for c fos,  **fosB** , c  **jun** , and junB. mRNAs for c fos,  **fosB** , c  **jun** , and junB were detected in the whole lens epithelium from the vicinity to the wound to the equator from 30 min to 8 hr post injury with their peaks after 30 min to 1 hr, but were no longer detected at 10 hr or later.  Ref: 11825018 Exp Eye Res, 2001
- The defect is secondary to an inability to phosphorylate CREB and to induce CREB dependent immediate early genes, including c  **jun** ,  **fosB** , fra2, and junB, which are required for cytokine gene induction.  Ref: 11739719 Mol Cell Biol, 2002
- In the lateral thalamus the repeated stimulation again failed to induce c Fos, but the expressions of  **FosB** , c  **Jun**  and Krox 24 were increased,.  Ref: 11744254 Neuroscience, 2001
